# Enzalutamide: A New Agent for the Prostate Cancer Treatment Armamentarium

**DOI:** 10.6004/jadpro.2013.4.3.7

**Published:** 2013-05-01

**Authors:** Stephanie B. McCutcheon

**Affiliations:** From Cancer Centers of the Carolinas, a department of the Greenville Hospital System, Greenville, South Carolina

Prostate cancer is one of the most common malignancies among men; it is estimated that 238,590 new cases will be diagnosed and approximately 29,720 deaths may be attributed to prostate cancer across the United States in 2013 (American Cancer Society, 2013). This disease is most frequently diagnosed in men over 65 years of age, with varying median survival depending upon the extent of the disease and response to therapies (National Cancer Institute, 2012). There are multiple types of treatment modalities currently available for consideration, dependent upon patient-specific factors; these options include active surveillance, surgery, radiation, hormone therapy, chemotherapy, and immunotherapy (National Comprehensive Cancer Network [NCCN], 2013).

As an androgen-dependent disease, prostate cancer may initially respond to therapies that reduce testosterone levels or inhibit androgen receptor binding. At some point, however, the disease may progress despite the use of orchiectomy or a luteinizing hormone-releasing hormone (LHRH)/gonadotropin-releasing hormone (GnRH) agonist, even in the setting of low serum testosterone (Scher et al., 2012). This condition is known as castrate-resistant prostate cancer. Androgen receptor signaling is a key factor in many metastatic castration-resistant prostate cancers; the androgen receptor may be overexpressed, mutated, and possibly activated by the androgens produced by tumor cells in progressive disease (Scher et al., 2010; Vogelzang, 2012).

Commonly utilized nonsteroidal androgen receptor antagonists, such as bicalutamide, work by competitive inhibition of androgens to their receptors. However, in addition to this antagonist activity these first-generation antiandrogens have also demonstrated some agonist activity (Tran et al., 2009). It is recommended that treatment with androgen receptor antagonists be used in combination with an LHRH/GnRH agonist in order to suppress androgen production (NCCN, 2013).

The development of disease resistance to current drugs underlies the need for additional treatment options for metastatic prostate cancer patients; enzalutamide (Xtandi) was developed in an attempt to improve antiandrogen agent options and to help overcome these mechanisms of resistance (Tran et al., 2009). Extending the use of hormone therapies for this patient population may have several benefits, such as maintaining a good quality of life, avoiding or delaying the need for utilization of chemotherapeutic agents, and providing therapy options after disease progression or intolerance to chemotherapy. There are relatively few efficacious chemotherapy options for this disease state, and the elderly population in particular may seek to avoid routine IV dosing and the possible serious side effects and complications associated with those drugs.

## Indication and Pharmacology

The indication for enzalutamide is treatment of patients with metastatic castration-resistant prostate cancer who have previously received docetaxel (Astellas Pharma US, 2012). Enzalutamide is an oral androgen receptor antagonist that exerts its action by affecting multiple steps in the androgen receptor signaling pathway. It does not lower androgen levels, but in a novel mechanism of action, it inhibits androgen receptor signaling in three different places: (1) blocks androgen from binding to androgen receptors through competitive inhibition, (2) inhibits nuclear translocation of androgen receptors, and (3) interferes with the interaction between DNA and androgen receptors (see Figure; Scher et al., 2012). In a drug-development study, enzalutamide also demonstrated a greater affinity for androgen receptors vs. bicalutamide without causing agonist activity (Tran et al., 2009).

**Figure 1:**
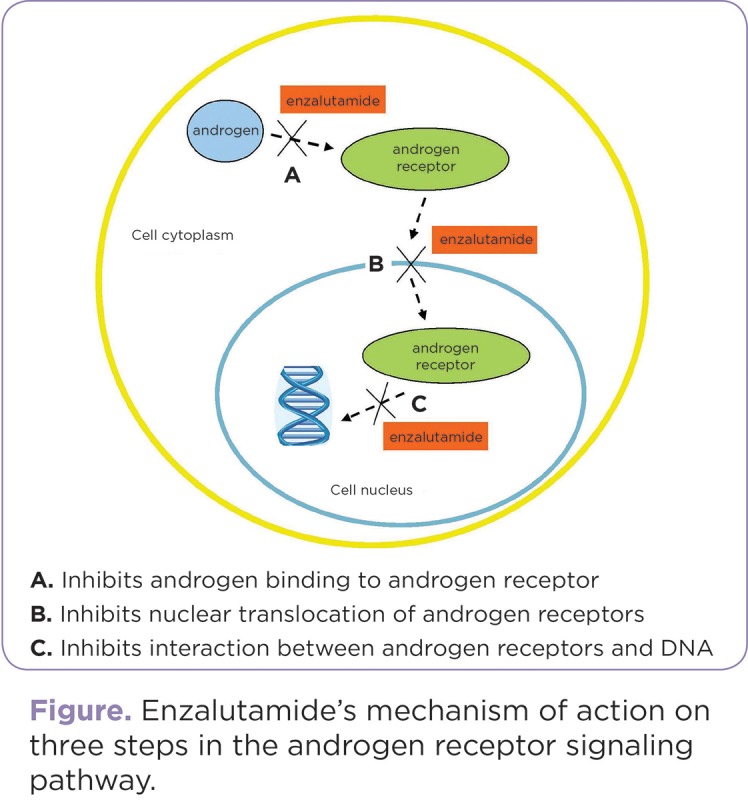
Figure 1. Enzalutamide’s mechanism of action on three steps in the androgen receptor signaling pathway.

## Clinical Studies

A phase I/II study administering enzalutamide in castration-resistant prostate cancer was conducted among 140 patients with or without a history of chemotherapy treatment (Scher, 2010). The primary objectives of the study were to assess pharmacokinetics, investigate safety and tolerability, and determine the maximum tolerated dose of the agent. Secondary objectives included assessing efficacy based on prostate-specific antigen (PSA) changes, soft-tissue and bone imaging, and time to disease progression. The most common grade 2 adverse events were fatigue, nausea, dyspnea, anorexia, and back pain. Adverse events that led to discontinuation of treatment by eight subjects were seizure, rash, fatigue, nausea/vomiting, and myocardial infarction; all of these events occurred at higher than the US Food and Drug Administration (FDA)-approved dose. There were notable decreases in PSA values among subjects, both with and without previous chemotherapy treatment. Imaging also demonstrated encouraging antitumor effects in soft-tissue and bone disease; median time to radiologic disease progression was 47 weeks (95% confidence interval [CI] = 34 wk–not yet reached).

The maximum tolerated dose for this study was defined to be 240 mg; however, the study group found that antitumor effects reached a plateau between the 150- and 240-mg doses and suggested that androgen receptor binding may be saturated with drug at plasma concentration levels consistently achieved by subjects at the 150-mg dosing level. The promising results of this early trial led to the development of a phase III study utilizing a dose of 160 mg per day.

The AFFIRM trial (A Study Evaluating the Efficacy and Safety of the Investigational Drug MDV3100) was an international, phase III, randomized, double-blind, placebo-controlled study that ultimately led to the drug’s approval by the FDA. The study population consisted of patients with metastatic castration-resistant prostate cancer, previously treated with at least docetaxel, and demonstrating progressive disease (Scher et al., 2012). The 1,199 enrolled participants were randomized 2:1 to receive either enzalutamide 160 mg orally once daily or placebo until disease progression, initiation of a new antineoplastic treatment, unacceptable toxicity, or withdrawal (Astellas Pharma US, 2012). All subjects continued androgen deprivation therapy, and concomitant usage of glucocorticoids was allowed but was not part of the required treatment protocol. The primary endpoint was overall survival, defined as the length of time from randomization to death from any cause; secondary endpoints included measures of response (PSA reduction, objective soft-tissue response, quality-of-life score) and measures of disease progression (time to PSA progression, radiographic progression-free survival, time to first skeletal-related event; Scher et al., 2012).

The AFFIRM trial was stopped early after a statistically significant overall survival benefit was seen for enzalutamide over placebo at a planned interim analysis after 520 death events. The median overall survival for enzalutamide was 18.4 months (95% CI = 17.3 mo–not yet reached) vs. 13.6 months (95% CI = 11.3–15.8 mo) for placebo; results at the interim analysis demonstrated a hazard ratio of 0.63 (95% CI = 0.53–0.75; * p* < .001) in favor of enzalutamide. A statistically significant benefit for enzalutamide vs. placebo was also seen among all of the secondary endpoints. Among the study subjects, 16% receiving enzalutamide and 18% receiving placebo discontinued the drug due to adverse events (Astellas Pharma US, 2012). Adverse events reported at a higher rate among the enzalutamide population included fatigue, diarrhea, hot flashes, musculoskeletal pain, headache, hypertension, and seizure. The trial authors allowed that there may have been predisposing factors in some of the subjects experiencing seizure, including brain metastases, brain atrophy, and other recently administered drugs (Scher et al., 2012).

## Dosing and Administration

The recommended dose of enzalutamide is 160 mg orally once daily, administered as four 40-mg gelatin capsules. It may be taken with or without food, but capsules should be swallowed whole; patients should not chew, crush, dissolve, or open the capsules. If a ? grade 3 toxicity or intolerable side effect is experienced, doses should be withheld for 1 week or until resolution to ? grade 2. When enzalutamide is resumed, it may be at the same or a reduced dose (120 or 80 mg) as necessary. If a patient requires the coadministration of a strong CYP2C8 inhibitor, the enzalutamide dose should be reduced to 80 mg once daily. It appears that there are no initial dose adjustments required for baseline mild to moderate renal or hepatic impairment; severe renal impairment, end-stage renal disease, and baseline severe hepatic impairment have not been assessed (Astellas Pharma US, 2012).

## Safety

In phase III clinical trial data, some adverse events seen at a higher rate among the enzalutamide population vs. those receiving placebo included fatigue, diarrhea, hot flashes, musculoskeletal pain, headache, peripheral edema, and seizure (Scher et al., 2012). Grade 3 or higher events were experienced in 47% of subjects receiving enzalutamide and in 53% of subjects receiving placebo (Scher et al., 2012; Astellas Pharma US, 2012).

Of the 800 participants in the phase III randomized trial receiving enzalutamide, 7 (0.9%) reported having a seizure from 31 to 603 days after drug initiation; there were no seizure occurrences reported for the placebo group. Resolution was reported upon discontinuation of therapy, and there is no clinical experience attempting to readminister enzalutamide. Patients with predisposing factors for seizure were excluded from the trial, so safety in this population is not known (Scher et al., 2012; Astellas Pharma US, 2012).

Other clinical trial adverse events highlighted by the manufacturer include infections, falls and fall-related injuries, and hallucinations. A total of 1% of subjects receiving enzalutamide vs. 0.3% receiving placebo died from infection or sepsis; 4.6% of enzalutamide patients vs. 1.3% of placebo patients experienced falls or fall-related injuries; and 1.6% of enzalutamide subjects vs. 0.3% of placebo subjects reported grade 1 or 2 hallucinations (Astellas Pharma US, 2012).

## Drug Interactions

Enzalutamide undergoes hepatic metabolism via CYP2C8 and CYP3A4, with CYP2C8 primarily responsible for the formation of an active metabolite, N-desmethyl enzalutamide. Plasma concentrations of enzalutamide may be altered by coadministration of strong or moderate CYP2C8 or CYP3A4 inhibitors or inducers (see Table), so they should be avoided if possible. A dose reduction of enzalutamide is suggested if it must be given along with a strong CYP2C8 inhibitor. Enzalutamide also may affect the plasma concentrations of other drugs, as it is a strong CYP3A4 inducer and a moderate CYP2C9 and CYP2C19 inducer (Astellas Pharma US, 2012).

**Table 1:**
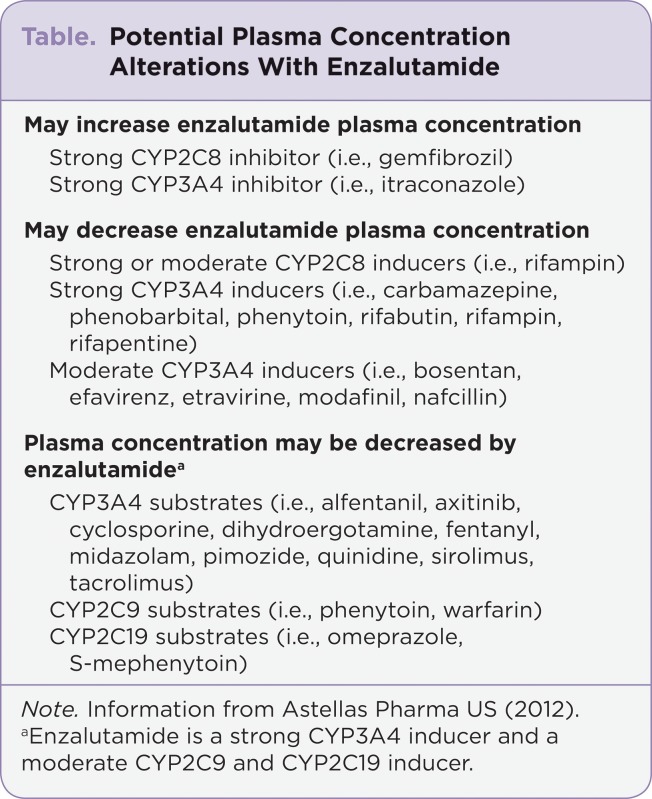
Table 1. Potential Plasma Concentration Alterations With Enzalutamide

## Implications

Enzalutamide gained FDA approval for use in metastatic castration-resistant prostate cancer following docetaxel chemotherapy after demonstrating promising interim analysis results in its phase III trial vs. placebo. It represents an important addition to the armamentarium of systemic treatment options for this progressive disease state. As it is incorporated into use, future questions to be answered include further elucidation of its places in therapy, the possibility of safe and efficacious combinations with other drug agents, and efficacy comparisons to existing therapies. There already exist several ongoing trials with enzalutamide attempting to answer some of these points: a phase III study in chemonaive progressive metastatic prostate cancer, a phase II study examining the combination with abiraterone and prednisone with bone metastatic castration-resistant prostate cancer, a phase Ib trial in combination with docetaxel, a phase II comparison with bicalutamide, and a phase II trial for neoadjuvant use in localized prostate cancer (ClinicalTrials.gov, 2012).

When initiating a patient on enzalutamide, some key considerations include conducting a thorough medication review to screen for potential drug interactions and continuing an LHRH/GnRH analog to maintain castrate levels of testosterone. Throughout the duration of therapy, routine monitoring includes complete blood counts, basic chemistry values, liver function tests, and assessing for signs and symptoms of adverse effects. Additional PT/INR monitoring may also be needed if the patient is on warfarin therapy.

Enzalutamide is currently available through specialty pharmacies. The manufacturer has an Access Services program established to assist patients with managing the drug expense; information is available at www.XtandiAccessServices.com.
